# Impact of transradial amputation and brachial plexus injury on vibrotactile sensation capacity of the upper extremity

**DOI:** 10.1186/s12984-026-02001-x

**Published:** 2026-05-16

**Authors:** L. A. Pardo, A. F. Schilling, M. A. Wilke, S. Dosen, J. Ernst, M. Markovic

**Affiliations:** 1 Hannover Medical School, Department for Trauma Surgery, Hannover, Germany; 2 Department of Trauma Surgery, Orthopaedics and Plastic Surgery, University Medical Center Göttingen, Göttingen, Germany; 3 Faculty of Life Sciences, Hamburg University of Applied Sciences (HAW), Hamburg, Germany; 4 Department of Health Science and Technology, Aalborg University, Aalborg, Denmark

**Keywords:** Vibrotactile sensation, Transradial amputation, Brachial plexus injury, Feedback, Psychometric assessment, Compensatory tracking

## Abstract

**Background:**

The sense of touch is fundamental for interacting with our surroundings, yet its restoration after sensory loss remains challenging. Haptic feedback, often delivered through vibrotactile stimulation, can help restore somatosensory information. While tactile perception on the arm is well characterized in healthy individuals, quantitative psychometric data are scarce for conditions involving severe peripheral disruption such as transradial amputation (TR) and brachial plexus injury (BPI), where denervation and cortical reorganization may reduce residual sensitivity.

**Methods:**

We systematically assessed the vibrotactile sensation capacity of the six relevant dermatomes (C3-T2) at the lower arm, upper arm, and shoulder regions of persons with TR and BPI. Psychometric tests were performed to determine the perception capacity (sensation threshold, just noticeable difference), and an online control task was conducted to assess the interpretation of feedback (delay and tracking error). The obtained results were compared to the benchmark data measured in healthy non-disabled individuals.

**Results:**

Within this small exploratory cohort, we found no significant differences in sensory capacity of persons with TR compared to the benchmark, in any outcome measure, and this was consistent across all arm segments. In contrast, persons with BPI demonstrated a significantly lower sensation perception in the distal regions (lower and upper arm), while the perception at the shoulder area was close to that of the benchmark. Unexpectedly, sensitivity was higher at the upper than the lower arm in persons with BPI, even though pan-plexus avulsion would be expected to cause complete sensory disconnection in both regions.

**Conclusions:**

The results suggest that vibrotactile sensation may remain partly preserved in proximal regions despite sensory impairment. Given the small sample and limited statistical power, these findings should be regarded as preliminary but may inform future designs of vibrotactile feedback interfaces.

**Supplementary Information:**

The online version contains supplementary material available at 10.1186/s12984-026-02001-x.

## Background

Sensory feedback is critical for movement planning and execution. This is consistent with sensory fibers grossly outnumbering motor fibers in the nerves innervating the human arm as shown recently [1]. Therefore, the loss of such feedback, due to injury and/or disease, can cause significant disabilities [2]. Experimental sensory deafferentation in healthy individuals confirms that even short-term disruption of afferent input reduces motor control precision in multi-digit tasks [3]. In prosthetic contexts, augmenting artificial feedback (e.g., audio or vibrotactile) improves performance and strengthens internal models compared to no feedback [4]. Hence, even when motor pathways are restored (for example via a robotic prosthesis), the absence or degradation of sensory feedback remains a critical limitation to functional recovery.

Recognizing the consequences of sensory loss, researchers have developed methods to provide artificial sensory feedback, using vibro-, electro-, and mechanotactile stimulation [5, 6]. These approaches have been employed to restore sensations in persons with amputation [7] and to facilitate therapy in other conditions with sensorimotor impairments [8]. The studies report that artificial sensory feedback can improve performance and subjective experience when using a prosthesis [9] and promote the recovery of sensory and motor functions in stroke subjects [10].

Vibrotactile stimulation is an attractive method to provide haptic feedback, as it is compact and easy to apply, hence suitable for wearable systems. It is also easier to apply compared to electrical stimulation, which can sometimes elicit discomfort or pain [11] if not properly calibrated. Vibrotactile stimulation activates fast-adapting (FA) mechanoreceptors in the skin, such as Meissner and Pacinian corpuscles, that respond to transient, high-frequency signals [12–14]. The skin’s capacity to perceive vibrotactile frequencies extends up to 1000 Hz, with Meissner corpuscles attuned to lower frequencies (10–100 Hz) and Pacinian corpuscles responsive to higher frequencies [15]. Impairments can disrupt these natural sensory pathways differently, which may affect the sensitivity of the participants to artificial stimulation. For instance, in nerve avulsion injuries, sensory input is directly lost due to nerve disconnection, resulting in notable perceptual deficits. In contrast, transradial amputations leave proximal nerves intact, but neural reorganization occurs, altering perceptual regions to form new sensory maps. Such conditions, therefore, can impact the patients’ capabilities to perceive and interpret artificial sensory feedback.

In persons with traumatic transradial amputation (TR), even without proximal nerve lesions, sensation on the residual limb changes post-amputation due to neural adaptations, such as the appearance of “phantom hand map” (PHM). Stimulating specific areas on the forearm can evoke sensations as if they originated from the missing hand. Notably, some studies have shown that tactile discrimination in PHM regions can be enhanced [16]. A similar phenomenon has been observed in persons with congenital amputation [17]. Complementing these findings, Van Gils et al. [18], investigated clinical sensibility of the residual limb using standardized measures of touch-pressure, stereognosis, and kinesthesia, and reported that stump sensibility was largely preserved relative to the unaffected limb, although reduced compared with healthy controls and influenced by prosthesis use. Together, these studies indicate that while global clinical sensibility at the residual limb may remain largely intact, localized or task-specific tactile processing can be altered or reorganized after amputation, underscoring the importance of quantitative, site-specific assessments for the design of sensory feedback systems.

For brachial plexus injuries (BPI), sensory losses vary with injury location. Proximal injuries often result in extensive sensory deficits, while two-point discrimination may remain in areas with partial innervation or overlapping dermatomes [19]. A comprehensive sensory map for BPIs is lacking and developing one is essential for an effective application of therapeutic feedback. BPIs disrupt motor, sensory, and autonomic fibers, causing dysfunction in the upper limb [20]. This sensorimotor incongruence often leads to neuropathic pain, such as phantom limb pain, due to abnormal signals from damaged axons [21–24].

In a previous study [25], we mapped vibrotactile sensitivity across the arm and shoulder in healthy able-bodied individuals. The findings showed that sensation thresholds (*ST)* vary by location, with higher thresholds at the shoulder. The Weber fraction *(WF)*, indicating the sensitivity to relative changes in stimulus intensity, remained consistent across the arm. There are only a few assessments of skin sensitivity in TR participants [17, 18], mostly focusing on the forearm, but no such studies in persons with BPI [26].

To provide functionally relevant and possibly therapeutic feedback, it is mandatory to understand how the sensations in the residual arm change proximally or distally from the traumatic injury or congenital deformity to the peripheral nerves. The small number of studies assessing the intrinsic sensory thresholds and perceptual capabilities of the full arm in both persons with TR and BPI using rigorous psychometric methods, presents a clear gap in the literature.

Here, we performed a comprehensive set of psychometric evaluations of tactile sensation on five persons with TR amputation, to assess proximal sensation capacities, and on six persons with pan-BPI (complete avulsion of all brachial plexus roots), to assess distal sensation capacities (here, proximal and distal refer to the place of injury). The tests included the determination of the *ST*, the just noticeable difference *(JND)*, and the perception of dynamically changing stimulation (online control using compensatory tracking task). The parameters thus obtained were compared with the reference values measured in able-bodied subjects, which define the benchmark [25].

## Methods

To compare the perception of vibrotactile stimulation in two distinct types of sensory disruption, five persons with transradial amputations (TR) (three with traumatic amputation: years since injury = 36.0 ± 24.2; two with congenital amputation) and six with pan-plexus brachialis injury (BPI; C5-Th1 avulsion with different reconstructions, years since injury = 13.0 ± 12.5) were recruited to participate in the study (Table 1). In the case of TR, congenital cases were analyzed descriptively and are visually distinguished in the figures (dashed lines) to illustrate their potential differences from traumatic cases. All subjects signed an informed consent form approved by the University Medical Center Göttingen ethics committee (Ethics Number: 26/6/20).

**Table 1:** Demographic and clinical characteristics of participants

Patient	Nerves affected	Age	Gender	Side of injury	Handyness	Year of injury	Years since injury	Phantom limb pain	Orthosis/prosthesis	Length stump
BPI 1	C5-Th1	28	M	Right	Right	2015	5	Yes	Sling	
BPI 2	C5-Th1	41	M	Right	Right	2020	0	Yes	Sling	
BPI 3	C5-Th1	50	M	Left	Right	2018	2	Yes	Sling	
BPI 4	C5-Th1	46	M	Left	Right	1989	31	No		
BPI 5	C5-Th1	53	M	Right	Right	2008	12	Yes	Sling	
BPI 6	C5-Th1	58	M	Left	Right	2008	12	Yes	Sling	
TR 1	Transradial	31	M	Left	Right	Congenital		No	Myo. Prosth.	8 cm
TR 2	Transradial	20	M	Left	Right	Congenital		No	Myo. Prosth.	7 cm
TR 3	Transradial	61	M	Left	Right	1980	40	Yes	Myo. Prosth.	11 cm
TR 4	Transradial	62	W	Left	Right	1962	58	Yes	Myo. Prosth.	6 cm
TR 5	Transradial	66	W	Left	Right	2010	10	Yes	Myo. Prosth.	18 cm

For each participant, affected nerves or amputation type, age, sex, affected side, year and chronicity of injury or amputation, phantom sensation, and use of assistive devices are reported. In TR participants, residual limb length and prosthesis type are included (Myo. Prosth. = Myoelectric prosthesis, EMG-controlled)

### Experimental setup

**Fig. 1:**
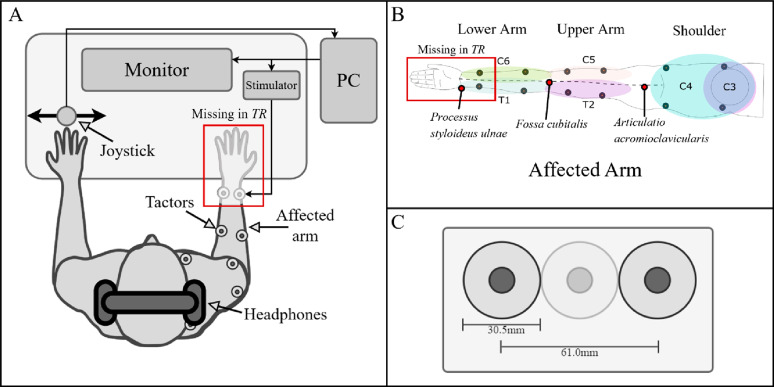
Experimental setup for both participants with amputation and participants with brachial plexus injury. The main difference is that the part of the limb was missing in the former group, as indicated by the box “Missing in TR”. **A** The experimental setup consisted of a PC for data recording and presentation of visual instructions on the monitor. The same PC also controlled tactile stimulation via a connected external stimulator that drove the tactors. A joystick served as the user command interface. The subject wore noise-canceling headphones on which white noise was played whenever the tactors were active to suppress the vibration-generated noise. **B**. A total of twelve tactors for persons with BPI, and ten tactors for persons with TR were placed on the arm and shoulder/neck of the affected side, reflecting the group-specific experimental configurations used for each condition. These are placed to stimulate the dermatomes (indicated as colored regions) innervated by the cervical spinal nerves C6, C5 proximally and distally (C6 only proximally for persons with TR), the thoracic spinal nerves T1 and T2 proximally and distally (T1 only proximally for persons with TR), and the cervical spinal nerves C3 and C4 ventrally and dorsally. We divided the limb into the lower arm (mainly innervated by T1 and C6), upper arm (mainly innervated by T2 and C5), and shoulder (mainly innervated by C3 and C4). **C** Additionally, we ensured that two tactors were always separated by a tactor-diameter, such that the minimum distance between the vibration centers (stimulation point) was at least 61 mm

The subject was seated comfortably in front of a desk with a computer screen during all experimental tasks, wearing active noise-cancelling headphones to prevent them from hearing the vibration. A modified joystick (springless) was connected to a PC via a USB port as a control interface (Fig. 1A). The PC controlled the output of the stimulation channels, which were attached to twelve high-end vibration motors based on voice-coil technology generating vibrations perpendicular to the skin (C2-tactor, Engineering Acoustics, Inc., USA; diameter: 30.5 mm [27]), with an optimal operating frequency of 230 Hz [28]. Notably, these tactors are linear resonant actuators (maximal displacement = 1.02 mm), thereby allowing for the modulation of both vibration frequency and intensity. However, the two parameters are partially coupled through a resonance effect. The amplitude values, which could be arbitrarily set via serial-port command interface (between 0 and 255 a.u.), are henceforth expressed in percentage of the maximal amplitude (i.e., 1.02 mm displacement). We controlled the tactors amplitude with a resolution of 0.39% (one step) while keeping their frequency fixed at their optimal operating value (230 Hz). The tactors were mounted inside custom 3D-printed holders that prevented mechanical interference between the fixation interface and the vibrating membrane. Each holder was secured to the skin using surgical tape, providing stable contact while allowing slight compliance to adapt to the arm’s contour. The tension of the tape was adjusted to ensure firm fixation with consistent pressure across participants but without overtightening, as excessive pressure could dampen the vibration amplitude and modify the perceived intensity. All tactors were placed on soft tissue regions, avoiding direct contact over bony landmarks to minimize variability in vibration transmission.

We divided the affected limb into three segments (Fig. 1B). The segments were evaluated individually, respecting the physiological organization in dermatomes, describing a skin area innervated by the afferent nerve fibers from the dorsal root of a single spinal root dermatome. The segments were defined as the lower arm (mainly innervated by T1 and C6), upper arm (mainly innervated by T2 and C5), and shoulder (mainly innervated by C3 and C4). The tactors were positioned based on consistent anatomical landmarks to correspond with the main dermatomes of each segment. On the lower arm, they were placed approximately at one-third and two-thirds of the distance between the processus styloideus ulnae and the armpit; on the upper arm, at one-third and two-thirds of the distance between the armpit and the acromioclavicular joint; and on the shoulder, at one-third and two-thirds of the half-distance between the acromioclavicular joints bilaterally. The ventral placements of C3 and C4 were preferred to avoid positioning tactors directly over the clavicle, where the very thin, soft-tissue layer can alter vibrotactile transmission and lead to less consistent perception (Fig. 1B). We investigated the vibrotactile sensation capacity within seven dermatomes (C3, C4, C5, C6, T1, and T2) of the arm-shoulder region of the affected side in persons with BPI and the side of amputation in persons with TR (Fig. 1B). The tactile sensations were elicited using twelve vibro-tactors for persons with BPI and ten in the case of persons with TR (Fig. 1B). They were placed in pairs of two on each of the dermatomes. In the case of persons with TR, given the variation in residual limb lengths (10 ± 4.85 cm) and to ensure methodological consistency, no tactors were placed on the distal lower arm (including distal T1 and distal C6). Three psychometric tests were used to quantify the subject’s response to vibrotactile stimulation. The exact methodology has been described in our previous study [25] and is briefly summarized in the next section. The benchmark data from that study were acquired in the same laboratory, by the same experimenter, using identical hardware (C2 tactors), calibration procedures, software, and task parameters as in the present work, and it served as the reference group in all between-group statistical tests

### Experimental protocol

The three psychometric tests were selected to provide a comprehensive evaluation of vibrotactile sensation by addressing distinct aspects of sensory perception. The tactile *ST* establishes the minimum detectable stimulus, characterizing each arm region’s basic sensitivity. The *WF* measures how well subjects can distinguish changes in stimulus intensity, which is crucial for creating nuanced feedback. Finally, the compensatory tracking task assesses the capability of continuous, real-time integration of sensory feedback to update motor output during an ongoing task, which is key for dynamic applications where timing and precision are important.

**Fig. 2:**
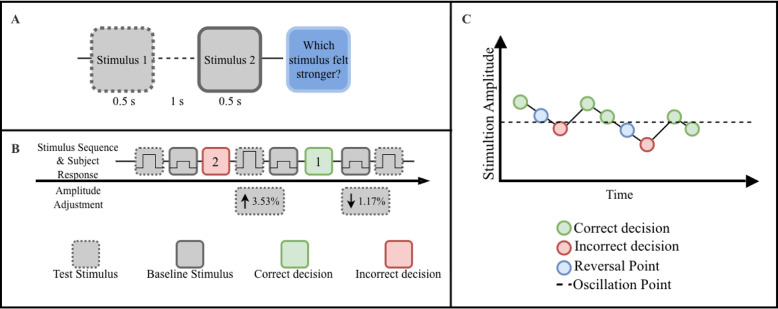
**A** Experimental protocol and psychophysical procedures. The baseline and test stimulus were presented in random order in a two-alternative forced-choice paradigm. Each stimulus was 0.5s long, and a break of 1 s was inserted between the stimuli. After the second stimulus, the participant had to decide (and select via the joystick) if the first or second stimulus had a higher amplitude (was stronger). **B** An schematic example of an adaptive sequence of trials. An incorrect decision (black rectangle) increased the amplitude of the test stimulus in the subsequent trial by 3.53% (nine discrete steps), whereas a correct decision (white rectangle) decreased it by 1.17%. **C** A schematic example of the staircase procedure used for threshold estimation. The amplitude of the stimulation was reduced after each correct decision and increased after an incorrect decision. A reversal point was defined as the last correctly identified amplitude preceding an incorrect decision. The oscillation point was computed as the average amplitude of ten reversal points (adapted from [25])

The method of limits was applied at each stimulation site to determine the *ST*. This standard psychophysical procedure determines the sensation threshold by gradually increasing stimulus intensity until the participant reports perception, thereby identifying the transition from no sensation to conscious detection. Here, a random vibrotactor was selected and initially activated at 0% amplitude at every repetition. The amplitude was then gradually increased in increments of 0.78% (two steps), with a 0.5-s pause between each step. The duration of the stimuli was set to last for 1.25 s. Because the stimulus amplitude was continuously increased and each burst lasted only briefly, adaptation effects were expected to remain minimal. This is consistent with recent evidence showing that vibrotactile adaptation occurs primarily after prolonged stimulation lasting several minutes, rather than during short intermittent bursts [29, 30]. Participants were instructed to verbally report the first instance when they consciously perceived the vibration. Each measurement was repeated three times per vibrotactor, and the average threshold value was recorded for each location to ensure reliability. Given that vibrotactile stimuli at maximum intensity did not induce discomfort, the upper limit of stimulation was set at 100%. The perceivable intensity range was therefore defined as [$\:{ST}_{i}$, 100%], where $\:{ST}_{i}$ represents the *ST* recorded at a given vibromotor location $\:i$.

If a participant exhibited a measurable *ST* at a given site, the *JND* in vibrotactile amplitude was determined using a two-alternative forced-choice task combined with a staircase procedure. One dermatome was stimulated repeatedly with a pair of vibrotactile pulses: a baseline stimulus (fixed at ST +15% of the perceivable range) and a variable test stimulus (Fig. 2A). The order of the two was randomized, and participants indicated which felt stronger by moving a joystick (left for first, right for second). The initial test intensity was set at ST +90% of the available range to avoid overstimulation and reduce adaptation effects. If the participant correctly identified the stronger stimulus, the intensity of the subsequent test stimulus was decreased by 1.18% (three steps). If the response was incorrect, the intensity was increased by 3.53% (nine steps) (Fig. 2B). This corresponds to the weighted staircase procedure with a step size ratio of 1:3 that identifies the *JND* at the success rate of 75% [31]. The process continued until ten reversal points were recorded, where a reversal was defined as an incorrect detection after a correct response. The point of oscillation was then computed as the average of reversal points, and the *JND* was calculated as the difference between the baseline and the point of oscillation (Fig. 2C). Finally, the *WF* at a given location *i* was computed using the following equation [32]: $\:{WF}_{i}=\frac{{JND\:}_{i}}{{b}_{i}}\times\:100$, where $\:{JND}_{i}$ and $\:{b}_{i}$ represent the *JND* and baseline stimulus at location $\:i$, respectively.

**Fig. 3:**
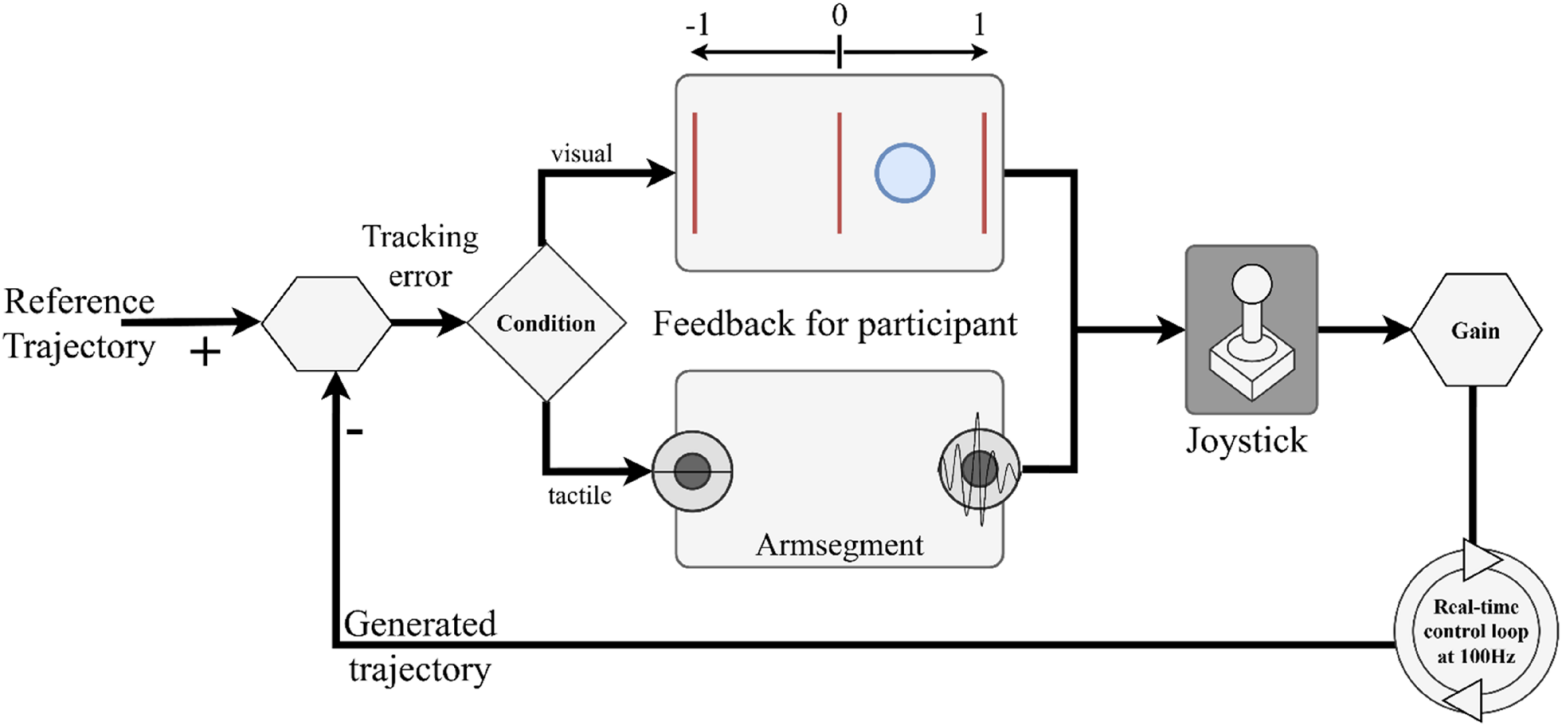
**A** Schematic illustration of the real-time control loop. The participant (human operator) is part of a dynamic system. The goal is to generate a signal with the joystick that minimizes the tracking error with respect to a predefined reference trajectory. The tracking error is communicated to the participant through tactile/visual feedback, depending on the condition. In the visual feedback, the onscreen circle conveys the sign and the magnitude of the error. In the tactile conditions, the active stimulator encodes the error sign and magnitude (adapted from [25])

Finally, we used a *compensatory tracking task* to study the subject’s ability to control and respond to continuous feedback across dermatomes. For this task, participants were required to track a 90-s pseudorandom multi-sine wave signal composed of nine frequency components ranging from 0.1 to 2 Hz. The tracking error, defined as the difference between the joystick position and the reference signal, was conveyed either visually or through vibrotactile feedback. In the visual feedback condition, the tracking error was displayed as a red circle moving along a horizontal axis on a computer screen, with a green vertical line marking zero error. Participants were instructed to adjust the joystick to keep the red circle aligned with this reference. Effectively, they moved the joystick to compensate for the current tracking error by pushing it in the opposite direction to the movement of the red circle around the reference line. This task is often used as a benchmark in human manual cybernetics to assess the quality of online closed-loop control [33]. In the vibrotactile feedback condition, the error was encoded spatially using a pair of tactors placed on different dermatomes within the same arm segment. One tactor indicated positive tracking error and the other negative, while the stimulation intensity was proportional to the error magnitude (with the normalized error mapped to the range 15–90% of stimulation intensity). Therefore, similarly as in the visual tracking condition, stimulation on one side of the arm indicated to the participant the need to move the joystick in the opposite direction, proportionally to the perceived stimulation intensity (Fig. 3).

Participants first performed training trials with visual feedback only, continuing until they demonstrated proficiency, typically after one to two attempts. They then completed ten trials using visual feedback alone, followed by three trials in the combined visual-vibrotactile condition to reinforce the association between visual and tactile cues. Once comfortable with vibrotactile feedback, participants trained with vibrotactile-only feedback for at least three trials or until achieving a tracking correlation coefficient of 0.6 or higher. For the final evaluation, participants completed ten additional trials per arm region using vibrotactile-only feedback. To minimize fatigue, a 1- to 2-min break was introduced between trials. To enable the spatial encoding of error, it was necessary that both dermatomes within a segment exhibited measurable *ST*. Therefore, if sensation could not be reliably measured at two points within a segment, the compensatory tracking task for that segment was omitted.

Some of the methods were modified with respect to the original study in healthy non-disabled participants [25] and adapted to the participant’s pathologies and their resulting impairments. In contrast to our previous study, where the joystick was operated with the stimulated hand, participants in the present study used their unaffected hand. This adjustment was necessary because persons with amputation without a prosthesis and BPI participants with limited motor or sensory function could not reliably control the joystick with the affected limb. The configuration ensured uniform task execution across participants and avoided motion-induced artifacts during stimulation. If no *ST* was measured at any tested point, as it reached saturation, it was defined to be 100% at this point. Accordingly, the *WF* was defined to be 100% at the same point, and no further experiments were performed using that vibration motor. The *ST* could physically not be measured on the distal lower arm of the person with amputation subgroup. However, we did not define it to be 100%, as it would distort the performance of this subject group in the proximal lower arm.

### Data analysis

For every individual subject, the *ST* of any of the twelve locations at the arm-shoulder region for BPI, and ten locations within the person with amputation subgroup, was estimated by averaging the data obtained from the three *ST* trials that were performed for that location. Then, the obtained values were averaged and compared across the three different upper extremity segments (lower and upper arm, and shoulder). Similarly, the average *JND* and *WF* were calculated across individual locations and arm-shoulder segments.

To analyse the performance in the compensatory tracking task, the correlation between the reference and the generated trajectory was identified as the peak of the cross-correlation function. Furthermore, the time delay between the target and the generated trajectory was estimated from the temporal location of the peak in the cross-correlation. After compensating for this delay by shifting the trajectory in time, the average absolute error between the generated and reference trajectory was calculated. Finally, the mean values of these three parameters (quantifying the shape similarity, average deviation, and delay, respectively) were calculated for each subject (and arm-shoulder segment) by averaging the outcomes of the ten compensatory tracking trials.

A one-sample Kolmogorov–Smirnov test revealed that none of the outcome parameters were normally distributed. Therefore, a Friedman test with a post-hoc Wilcoxon rank-sum test was used to detect statistical differences between different arm-shoulder regions within each participant group. Additionally, Kruskal–Wallis tests combined with post-hoc Wilcoxon rank-sum tests were used to compare each participant group (BPI and TR) separately against the healthy benchmark. All statistical tests were corrected for multiple comparisons using the Bonferroni-Holm correction. The statistical difference threshold was set to 0.05. All results are presented as “median [interquartile range (IQR)]”.

For each pairwise comparison, effect sizes were calculated as Cliff’s Δ to quantify the magnitude and direction of group differences and presented as “Δ [95% CI]” in the supplementary materials Table S1. Ninety-five-percent confidence intervals (CIs) for group medians were obtained by bootstrap resampling (10 000 iterations, bias-corrected and accelerated) and presented as “[95% CI]” in the supplementary materials Table S1.

### Post-hoc power analysis

Given the non-normal distribution of the data, all between-group comparisons were performed using nonparametric tests (Kruskal–Wallis followed by the Wilcoxon rank-sum). To quantify the sensitivity of this design, we conducted a post-hoc power analysis for the Wilcoxon rank-sum test (two-tailed, α = 0.05) using the noncentral U distribution. For comparisons between the transradial amputation group (*n* = 5) and the benchmark (*n* = 10), statistical power was 0.13 for a medium effect (Cohen’s d ≈ 0.5), 0.25 for a large effect (d ≈ 0.8), and 0.50 for a very large effect (d ≈ 1.2). Comparable values were obtained for the BPI group (*n* = 6). As expected, due to the low sample size, the present study is underpowered and should be interpreted as exploratory. Nevertheless, both conditions (BPI and upper limb amputations) have a generally low prevalence, and the number of participants in the literature is comparable to the sample size used in the present study [34, 35] In addition, this is the only study that included both target groups, thereby providing preliminary insights into their sensory status.

## Results

**Fig. 4:**
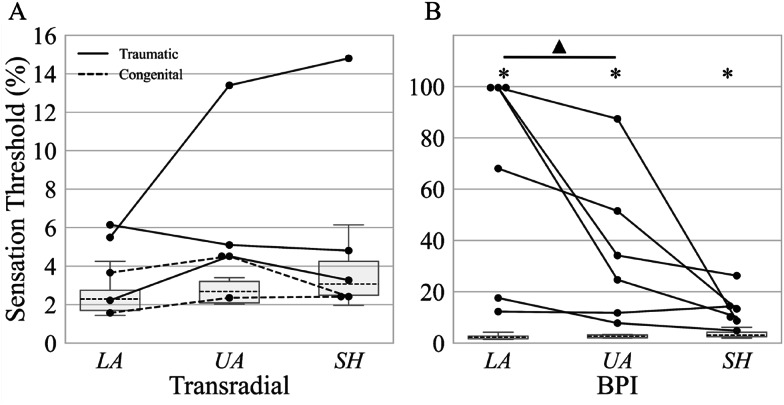
Sensation thresholds for the lower arm (LA), upper arm (UA), and shoulder region (SH), expressed in percent of the maximum stimulation amplitude for **A** persons with transradial amputation (TR) and **B** persons with brachial plexus injury (BPI). The individual dots represent data points. The dashed, horizontal lines indicate the median of the benchmark data obtained in healthy nondisabled participants [17]. Asterisks (*) denote statistically significant differences (*p* < 0.05) for a given segment when comparing persons with TR and BPI, to the benchmark. Triangles above a horizontal bar indicate statistically significant differences between the connected arm segments within the same population (persons with BPI or TR). Note that the y axes have different scaling for TR and BPI

The sensation capacity on the lower arm, upper arm, and shoulder region of able-bodied participants was measured in our previous work and adopted in the present study as the sensation benchmark (normal sensitivity) [25]. Herein, we show the summary results for the sensation capacity of BPI and TR participants and compare them to the benchmark. For clarity, it should be noted that the y-axis scaling differs between TR and BPI panels in Fig. 4, reflecting the substantially different dynamic ranges of sensation thresholds across the groups. In addition, absent data points in distal arm regions of the BPI group indicate a lack of detectable sensation at maximal stimulation rather than missing measurements. For completeness, all results are reported in tabular form in the supplementary material attached to this manuscript.

Importantly, the comparisons between the groups should be considered mostly qualitative since the lack of significance can be due to the limited statistical power, as explained in the Methods.

### The sensation threshold

In the BPI group, *ST* was significantly higher at the lower arm compared to the upper arm (*p* < 0.01), while no significant differences were found between the lower arm and the shoulder or between the upper arm and the shoulder after correction (Fig. 4B). Nevertheless, there is clear trend that the sensitivity was the highest at the shoulder, and the lack of significance is likely due to a low sample size. Three BPI participants maxed out the *ST* (100%) when it was measured at the lower arm. In the TR group, no significant differences were observed in *ST* values between any of the three measured segments.

When comparing these results to the benchmark, persons with BPI exhibited significantly higher *ST* across all measured regions (*p* < 0.001, lower arm; *p* < 0.001, upper arm; *p* < 0.001, shoulder). In contrast, TR showed no significant differences in *ST* compared to the benchmark in any of the segments (Fig. 4A).

### The Weber fraction

**Fig. 5:**
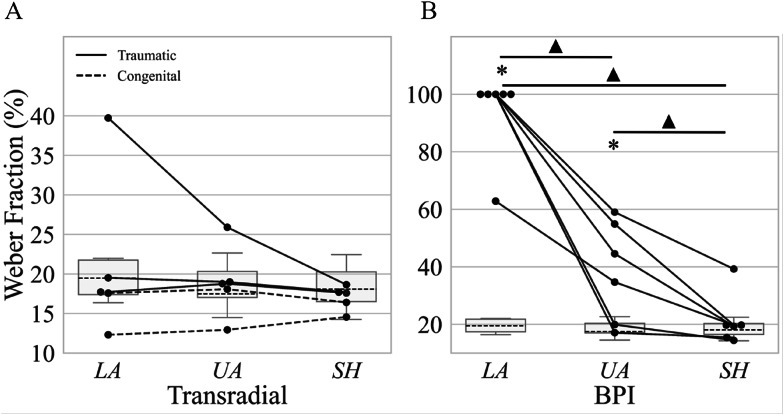
Weber fraction for the lower arm (LA), upper arm (UA), and shoulder region (SH), expressed in percent of the maximum stimulation amplitude for **A** persons with transradial amputation (TR) and **B** persons with brachial plexus injury (BPI). The annotation is the same as in Fig. 4. Note that the y-axes have different scaling for TR and BPI

Regarding the *WF*, only one of the six persons with BPI was able to reliably perceive differences in stimulus intensity at the lower arm, resulting in a median *WF* of 100% (Fig. 5B). This was significantly higher than the *WF* obtained for the upper arm (*p* < 0.05) and shoulder (*p* < 0.05). Additionally, the *WF* in persons with BPI was significantly higher at the upper arm compared to the shoulder, with the values twice as high in the former segment (*p* < 0.05). In the TR group, no significant differences in *WF* were observed between any of the arm segments.

When comparing these results to the benchmark data, the *WF* obtained in persons with TR was within the benchmark range in most cases, consistently remaining below 20% and with no significant differences with respect to the benchmark for any of the tested segments (Fig. 5A). In contrast, persons with BPI exhibited significantly worse *WF* in comparison to the benchmark at the lower arm (*p* < 0.05). Moreover, *WF* at the upper arm was also significantly higher in BPI compared to the benchmark, with the values approximately twice as high (*p* < 0.001). The *WF* at the shoulder, however, was similar to that of the benchmark (Fig. 5A).

### Compensatory tracking task

As reported in the previous section (Fig. 5B), only one of the persons with BPI was able to perceive differences in vibration stimuli at the lower arm. Therefore, the compensatory tracking task was not performed for this region in the group of persons with BPI. Additionally, one participant with BPI discontinued participation before completing the compensatory tracking task, therefore, only five individuals are represented in this section.

**Fig. 6:**
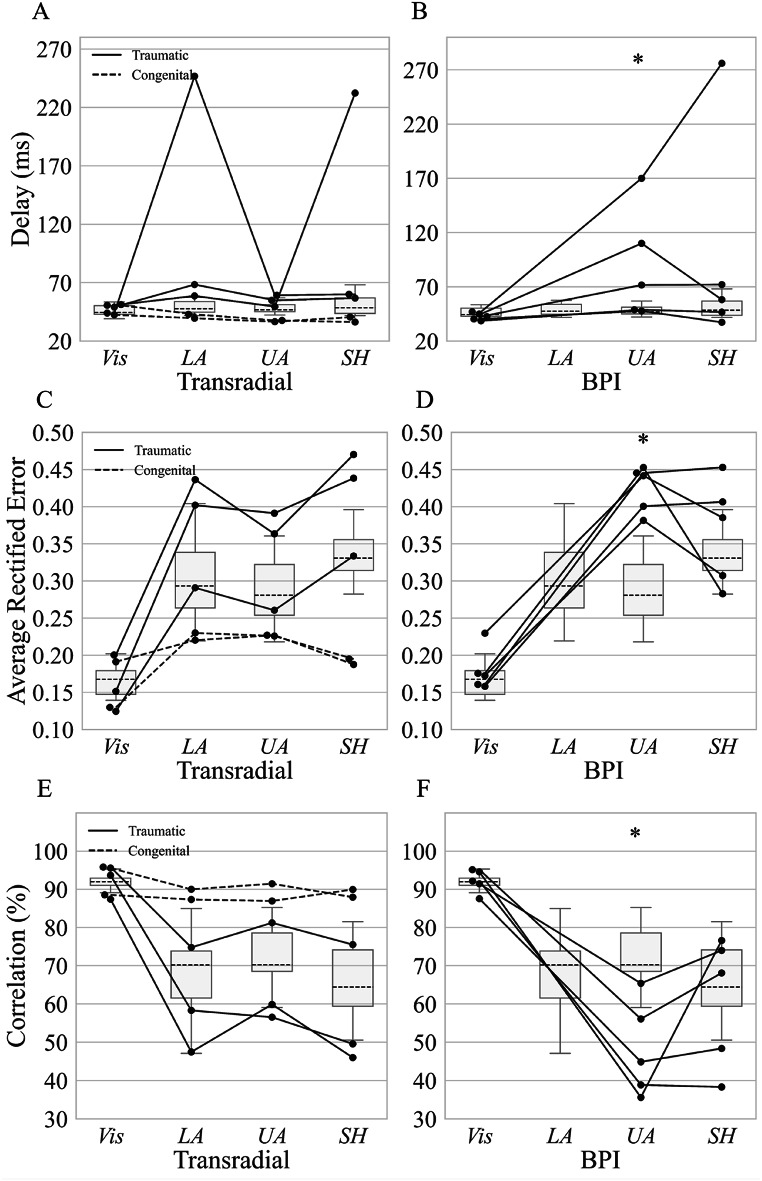
Performance outcomes during the compensatory tracking task with tactile feedback, evaluated at the lower arm (LA), upper arm (UA), and shoulder (SH) regions. Delay between the participant’s joystick signal and the reference trajectory (reported in milliseconds [ms]) in **A** persons with transradial amputation (TR) and **B** persons with brachial plexus injury (BPI). Average absolute error (normalized, unitless) representing the mean absolute deviation from the reference trajectory in **C** persons with transradial amputation (TR) and **D** persons with brachial plexus injury (BPI). Correlation coefficient (reported as percentage [%]) indicating the dynamic similarity between participant-generated and reference signals in **E** persons with transradial amputation (TR) and **F** persons with brachial plexus injury (BPI). “Vis” denotes the visual feedback condition used as a baseline. Horizontal dashed lines indicate group medians; each dot represents one participant. Asterisks (*) indicate statistically significant differences (*p* < 0.05) between groups or conditions. Annotations are consistent with Fig. 2

No significant differences in delay were observed between either persons with TR and the benchmark, nor persons with BPI and the benchmark, when using visual feedback. However, when only tactile feedback was provided, the delay was significantly higher in persons with BPI for the upper arm compared to the benchmark (71.6ms [61.2ms] vs. 47.7ms [6.0ms]; *p* < 0.05) (Fig. 6B). In the same region, persons with BPI also exhibited a higher average absolute error (0.44 [0.04] vs. 0.28 [0.06]; *p* < 0.001) (Fig. 6D) and a lower correlation (44.9% [17.2%] vs. 70.2% [10.1%]; *p* < 0.001) (Fig. 6F). No significant differences were found between persons with BPI and the benchmark for the shoulder region in any of the outcome measures. The results for persons with TR were not significantly different compared to the benchmark in any of the tests. In either of the two groups, there was no significant difference between the tested arm segments. While no statistical comparison was performed between persons with TR and BPI due to the low number of participants, the observed trend suggests that persons with BPI performed similarly to persons with TR and able-bodied benchmark at the shoulder segment.

## Discussion

This study was performed to understand how persons with BPI and TR experience sensory input compared to a benchmark defined by results from healthy able-bodied individuals. Because both clinical populations are rare and the protocol was extensive, the study was necessarily limited in sample size (persons with TR = 5, persons with BPI = 6). Accordingly, all results should be regarded as preliminary and descriptive rather than confirmatory. Further, because the tracking task was controlled with the unaffected hand, the affected limb remained at rest during sensory testing. The reported thresholds therefore reflect the sensory capacity of the stimulated regions in isolation. In practical prosthesis use, vibrotactile feedback would typically be delivered to the same limb that also provides myoelectric control, where residual muscle activity and attentional demands may influence perceptual clarity. The thresholds reported here may thus represent an upper-bound estimate of tactile sensitivity, and this difference should be considered when comparing the present results to the benchmark or when translating them to applied prosthetic-control scenarios.

### Tactile sensitivity appears largely preserved in persons with transradial amputation

We found no significant differences between persons with TR and the benchmark in any outcome metrics regarding sensory capacity. While some studies have reported altered sensory function in persons with TR amputation, particularly in phantom hand map regions [16, 17], our results suggest that vibrotactile sensation at the residual limb may remain comparable to that of able-bodied individuals, aligning with the findings of Van Gils et al. [18]. Importantly, this pattern reflects a group-level tendency relative to the benchmark and does not imply uniform preservation across individuals. This is further underscored by the inter-individual variability visible in the data of participants with amputations. However, this is an important practical implication indicating that sensory capacity in the residual limb may be largely retained after amputation, which can be used to implement effective sensory substitution systems to provide artificial somatosensory feedback from the prosthesis to the user. Furthermore, the lack of substantial difference between persons with TR and the benchmark indicates that such systems can be tested in non-disabled individuals, initially, to identify a promising first design, which then needs to be refined by testing in target users. Importantly, the present study shows that this holds not only for the perception of stimulation (*ST* and *WF*) but also for the interpretation and the use of feedback during online control (compensatory tracking task). However, the figures show occasional outliers where sensitivity was worse compared to the benchmark, and hence, the translation of the insights from the tests in non-disabled participants might not hold in all cases. A reason for this heterogeneity might be our cohort of participants with TR comprising both congenital and traumatic cases. Persons with a congenital amputation usually lack phantom-hand experiences and show redistributed or atypical somatosensory representations [36, 37], whereas individuals with traumatic amputation commonly retain phantom-hand maps, and cortical reorganization may be further shaped by chronic phantom pain or long-term prosthesis use [38]. Additionally, the two congenital participants (ages 20 and 31) were markedly younger than the traumatic amputees (ages 61–66) and did not report phantom limb pain (Table 1). Age is a well-established determinant of vibrotactile sensitivity, with younger adults showing substantially lower sensation thresholds and finer discrimination [39]. This likely contributed to the comparatively better sensitivity observed in the congenital participants, in some cases approaching the lower range of the benchmark group. These differences likely influence tactile perception at the residual limb and need to be considered.

Despite these sources of individual variability, the overall pattern of responses within the group of persons with TR was remarkably consistent across arm segments, suggesting that tactile feedback could be provided at proximal segments without substantial loss of sensation quality, enabling flexible design choices that bypass existing bottlenecks, such as limited space for embedding feedback devices directly in the socket [40]. While adjustments may be necessary to adapt sensory feedback when the stimulators are placed further away from the prosthesis, evidence shows that users can adapt over time, integrating new sensory inputs with reduced cognitive load [41]. This is a valuable insight and a viable design option, specifically in those cases where residual stump length and injury conditions, like scars or short residual limbs, impose significant special limitations for embedding a tactile interface in the prosthesis socket. However, it needs to be considered that displacing the tactile feedback more proximally leads to a system that would not be self-contained.

Moreover, the delays observed during the compensatory tracking task showed that the cognitive processing of stimuli and the time taken to execute motor commands are not substantially influenced by the proximity of the stimulation site to the spinal cord or its starting point on the arm. This further indicates that persons with TR can utilize feedback devices with the same level of cognitive effort and efficiency across all dermatomes of the arm and shoulder. Assuming that visual tracking has a low cognitive effort and a high efficiency, reaching delays lower than 50 ms, the results obtained using tactile stimulation are encouraging since tactile feedback increased the delay by only 10 ms on average, without a significant difference compared to the visual feedback. Importantly, the compensatory tracking task represents a highly controlled laboratory paradigm designed mainly to probe the interpretation and temporal use of continuous feedback signals. It does not model the full complexity of prosthetic manipulation, as the stimulated limb remained at rest. During prosthesis control, however, the user activates muscles to produce motor commands and the residual limb is also exposed to load-dependent interactions (e.g., lifting a heavy object). Consequently, the similar tracking performance obtained in the present study should be interpreted as evidence for preserved feedback interpretation under controlled conditions, rather than as a direct proxy for functional usability during real-world prosthesis control. Also, visual inspection of the trajectories indicates that persons with TR and the benchmark participants track the reference signal with very similar dynamics. Both groups show rapid corrections and only brief deviations during rapid changes of the target, with little evidence for excess jitter or delayed convergence (Figure S1 C-F). However, this does not necessarily translate to immediate or intuitive use of vibrotactile feedback during prosthesis control. Vibrotactile cues are normally non-somatotopic (except if they are delivered to the PHM) and therefore require sensorimotor learning to associate the artificial signal with the corresponding mechanical or visual event [42].

### Tactile perception in persons with brachial plexus injury substantially worse in the distal arm segments

It was expected that persons with BPI would show diminished sensory function at the lower arm due to the extent of nerve injury; indeed, four out of six individuals did not perceive any stimuli in the distal segments of the lower arm (distal C6/T1) even when the tactors were activated at the full intensity. This pattern aligns with prior reports that BPI elevates sensation thresholds and can even alter somatosensory function bilaterally due to central reorganization [43].

However, we initially anticipated a more uniform decline in sensory perception across all brachial plexus-innervated dermatomes. Contrary to this expectation, ST and WF at the upper arm were substantially lower than at the distal arm, indicating a relative preservation of proximal sensation. While counterintuitive given the typical proximal root involvement in pan-plexus avulsions, retained or partially restored proximal sensibility has been described in population subsets of persons with BPI, consistent with heterogeneity arising from variable surgical reconstruction and reinnervation. This unexpected finding suggests that proximal sensory capacity in persons with BPI may be higher than often assumed, at least in some individuals, despite severe injury patterns [44]. Several mechanisms may contribute to this residual proximal sensitivity. Overlapping dermatomes between adjacent cervical roots (e.g., C4–C5 overlap), collateral sprouting of intact afferent fibers, or cortical reorganization within the primary somatosensory areas following deafferentation [45]. Such compensatory mechanisms may restore limited tactile input even after complete root avulsion. Historically, these subtle residual functions have often gone unnoticed because most sensory assessments in persons with BPI rely on coarse clinical tests (e.g., touch or pinprick), rather than quantitative psychophysical measures that detect small perception gradients. Beyond describing segment-dependent differences, these psychophysical measures provide a device-independent characterization of residual sensory capacity. Sensation thresholds and Weber fractions directly constrain how many discriminable intensity levels can be meaningfully encoded at a given site, and thus offer practical input for selecting feedback locations and mapping the resolution of stimulation. Such explicit quantification has been largely missing in BPI research and strengthens the translational relevance of our findings.

The shoulder region, which is supplied by the cervical plexus C3 and C4 nerve roots, indicates preserved sensory perception comparable to that of the benchmark (apart from somewhat higher *ST*). This finding aligns with our expectations, as this area is typically unaffected by brachial plexus injuries. This was further supported by the results of the compensatory tracking task. None of the persons with BPI could perform the task at the lower arm due to the lack of detectable sensation. When tactile feedback was applied to the upper arm, participants showed significantly impaired tracking performance compared to the benchmark group: delays were longer, errors higher, and the correlation with the target signal was markedly reduced. This functional deficit likely reflects the reduced tactile resolution in this region, as indicated by the increased *ST* and *WF*. In contrast, tracking performance at the shoulder was statistically comparable to the benchmark group, indicating that this region may offer relatively preserved perception and sufficient temporal control for potential feedback integration, supporting the practical relevance of proximal regions, particularly the shoulder, as candidate sites in appropriately selected individuals for implementing sensory feedback in persons with BPI. Importantly, this conclusion is derived from heterogeneous individual responses and should not be interpreted as evidence that proximal or shoulder-level sensory preservation is present consistently in all individuals with brachial plexus injury. Rather, it highlights the shoulder as a potential feedback site in selected cases, contingent on individual sensory assessment. However, when visually inspecting the curves, persons with BPI display less efficiently corrected deviations from the target trajectory, resulting in a slower return toward equilibrium (Figure S1 A, B). While the differences are subtle at the level of brief 10-s excerpts, they are consistent with the quantitative findings showing reduced tracking accuracy in BPI.

At the same time, sensory function varied considerably across individuals. Some participants retained perception in proximal regions, while others exhibited near-total sensory loss throughout the limb. This was expected, as variability of cutaneous sensibility with injury level and recovery status is well documented, including cohorts of persons with pediatric/obstetric BPI showing segment-dependent sensory deficits [46] and adult cohorts with central and peripheral contributors [43]. Consequently, it suggests that a standardized approach to sensory feedback for persons with BPI may not be optimal, as residual sensory function depends on individual recovery processes and injury-specific factors. Alternatively, feedback interfaces for persons with BPI could be implemented in the shoulder region, as the results showed that its sensory capacity was comparable to that of the benchmark.

Although the application of sensory feedback in persons with BPI rehabilitation is less established than in persons with amputation, emerging evidence indicates its potential benefits. For instance, integrating sensory feedback through modalities like virtual reality and robotics has shown promise in facilitating upper extremity pain management and sensory recovery in persons with BPI [47]. Additionally, neuromuscular electrical stimulation has been utilized for motor and sensory re-education in persons with brachial plexus injuries, demonstrating excitation of both motor and afferent sensory nerve fibers [26]. These findings suggest that implementing sensory feedback interfaces in preserved regions, such as the shoulder, could support rehabilitation efforts by enhancing sensory input and promoting functional recovery.

### Study limitations

While this study provides valuable insights into the sensory capacities of persons with BPI and TR, the findings are limited by the small sample size, which restricts the ability to generalize these results to the broader population.

A limitation of the present study is that phantom hand maps (PHMs) were not systematically assessed. Although some participants with TR mentioned occasional referred sensations, these were not recorded in a structured manner, and no conclusions can be drawn regarding the relationship between PHMs and vibrotactile sensation in this cohort.

Additionally, all participants with BPI in this study presented complete root avulsion (C5–T1), representing pan-plexus injuries. However, the type and timing of surgical reconstructions varied across individuals, reflecting the intrinsic heterogeneity of this clinical population (see Table 1). Such variability in surgical history, as well as in the degree of nerve regeneration and recovery stage, may contribute to the observed inter-individual differences in sensory performance and limits thereby limit the ability to draw stratified or generalizable conclusions. However, this reflects the clinical reality of the population in which the sensory feedback will be applied, and hence, the insights obtained in the present study are still practically relevant. Further research with larger sample sizes and a diverse range of injury types and recovery stages is needed to validate these findings and explore the full spectrum of sensory adaptations in both persons with BPI and persons with TR amputation.

Since the contact pressure between the tactor and the skin was adjusted manually, small discrepancies are possible between the measurement sites (though the experimenter gave their best to minimize any inconsistencies). Nevertheless, this is a standard approach in the literature [48, 49] and also reflects the way vibromotors are applied when conveying feedback, thereby providing practically meaningful results.

In addition, no standardized assessment of mental workload was conducted during the third experiment. The participants were trained to a stable performance level before data collection and rest periods were enforced between trials, as well as whenever participants demanded it or the experimenter noticed the signs of fatigue, which is the usual approach in the application of the compensatory tracking task [50, 51]. Future studies should include standardized workload questionnaires and learning-curve analyses to characterize cognitive demands and training during vibrotactile feedback use [52] This, however, would be most relevant when investigating the use of vibrotactile feedback within the clinical context (e.g., prosthesis use in TR).

Variations in amputation-related factors, such as skin grafts and flap coverages, were not specifically controlled in this study, yet they may influence sensory perception [53]. Reduced sensation in grafted areas or polyneuropathies induced by tumor-related chemotherapy could alter vibrotactile sensation, potentially leading to differences in sensory thresholds or response patterns compared to both the benchmark and participants with traumatic amputations. Such variations could pose challenges in placing tactile actuators on the distal residual limb, particularly when covered by a prosthetic socket. Similarly to persons with BPI, it is well known that persons with amputation represent a heterogeneous group, as the condition of the residual limb depends on the etiology but also surgical procedures. The properties of the residual limb could be assessed using clinical photography or ultrasound-based skin thickness measurements [54], and it would be relevant to investigate how these factors affect the sensitivity. This was, however, outside the scope of the present study and remains to be addressed in future work.

Two participants with TR amputation exhibited unusually long response delays during the compensatory tracking task at specific dermatomes. However, the statistical comparisons against the benchmark remained non-significant even when these data were included. Nonetheless, these deviations point out to potential inter-subject variability that should be further investigated on a larger samples with formal sensitivity analyses to evaluate the robustness of these findings.

Lastly, the present study employed a single tactor type (C2-tactor, Engineering Acoustics Inc.) operating at a fixed frequency of 230 Hz, primarily targeting Pacinian corpuscles. Consequently, frequency-dependent aspects of tactile perception were not captured, particularly in participants with altered cutaneous innervation.

## Conclusion

This study systematically examined how upper-limb sensorimotor impairments influence vibrotactile sensation across different arm segments in persons with brachial plexus injury (BPI), transradial (TR) amputation, and the benchmark (healthy controls). Within this small exploratory cohort, participants with TR amputation exhibited largely preserved sensory perception proximally to the amputation site, indicating that sensory feedback could be delivered to the intact skin areas above the socket, as a practical alternative when direct socket integration is compromised. In persons with BPI, sensation was markedly reduced in the distal arm but appeared partly preserved at the upper arm and shoulder. These preliminary observations suggest that proximal regions could serve as alternative stimulation sites for implementing sensory feedback in rehabilitation. The study provides a unique insight into the sensory status of these target groups obtained on a small sample, reflecting the overall low incidence of these conditions. Nevertheless, the results provide initial evidence that may guide the design of more flexible, personalized feedback systems in upper-limb neuroprosthetics.

## Supplementary Information

Below is the link to the electronic supplementary material.

Supplementary Material 1.

Supplementary Material 2.

## Data Availability

Data is provided within the manuscript or supplementary information files.

## Abbreviations

TR: Transradial
BPI: Brachial plexus injury
LA: Lower arm
UA: Upper arm
SH: Shoulder
ST: Sensation threshold
WF: Weber fraction
