# Supplementary material for: Impact of transradial amputation and brachial plexus injury on vibrotactile sensation capacity of the upper extremity
Source: J Neuroeng Rehabil. 2026 May 16;23:175. doi: 10.1186/s12984-026-02001-x (PMC13220424; doi:10.1186/s12984-026-02001-x)
Supplement: Supplementary file 1 — Supplementary Material 1. [file 12984_2026_2001_MOESM1_ESM.docx]

**7. Supplementary materials**

**7.1. Sensation threshold**

| **Experiment 1, Sensation threshold: Comparisons between participants** | | | | | |
| --- | --- | --- | --- | --- | --- |
| **Population** | **Segment 1** | **Segment 2** | **median[IQR] 1 (%)** | **median[IQR] 2 (%)** | **p-value** |
| BPI | Lower arm | Upper arm | 83.8 [69.4] | 29.4 [32.1] | 0.0313 |
|  | Lower arm | Shoulder | 83.8 [69.4] | 11.8 [5.1] | 0.0625 |
|  | Upper arm | Shoulder | 29.4 [32.1] | 11.8 [5.1] | 0.0625 |
| TR | Lower arm | Upper arm | 3.7 [3.3] | 4.5 [0.6] | 0.3125 |
|  | Lower arm | Shoulder | 3.7 [3.3] | 3.3 [2.4] | 1.0000 |
|  | Upper arm | Shoulder | 4.5 [0.6] | 3.3 [2.4] | 0.6250 |

| **Experiment 1, Sensation threshold: Comparisons between populations** | | | | | |
| --- | --- | --- | --- | --- | --- |
| **Population** | **Segment 1** | **Segment 2** | **median[IQR] 1 (%)** | **median[IQR] 2 (%)** | **p-value** |
| **Lower arm** | AB | BPI | 2.3 [1.0] | 83.8 [69.4] | 0.0015 |
|  | AB | TR | 2.3 [1.0] | 3.7 [3.3] | 0.3173 |
|  | BPI | TR | 83.8 [69.4] | 3.7 [3.3] | 0.0062 |
| **Upper arm** | AB | BPI | 2.7 [1.1] | 29.4 [32.1] | 0.0015 |
|  | AB | TR | 2.7 [1.1] | 4.5 [0.6] | 0.0719 |
|  | BPI | TR | 29.4 [32.1] | 4.5 [0.6] | 0.0176 |
| **Shoulder** | AB | BPI | 3.1 [1.8] | 11.8 [5.1] | 0.0032 |
|  | AB | TR | 3.1 [1.8] | 3.3 [2.4] | 0.6407 |
|  | BPI | TR | 11.8 [5.1] | 3.3 [2.4] | 0.0679 |

**7.2. Weber fraction**

| **Experiment 2, Weber Fraction: Comparisons between participants** | | | | | |
| --- | --- | --- | --- | --- | --- |
| **Population** | **Segment 1** | **Segment 2** | **median[IQR] 1 (%)** | **median[IQR] 2 (%)** | **p-value** |
| BPI | Lower arm | Upper arm | 100.0 [0.0] | 39.6 [28.7] | 0.0313 |
|  | Lower arm | Shoulder | 100.0 [0.0] | 19.5 [3.3] | 0.0313 |
|  | Upper arm | Shoulder | 39.6 [28.7] | 19.5 [3.3] | 0.0313 |
| TR | Lower arm | Upper arm | 17.7 [1.9] | 18.8 [0.9] | 1.0000 |
|  | Lower arm | Shoulder | 17.7 [1.9] | 17.6 [1.3] | 0.4375 |
|  | Upper arm | Shoulder | 18.8 [0.9] | 17.6 [1.3] | 0.3125 |

| **Experiment 2, Weber Fraction: Comparisons between populations** | | | | | |
| --- | --- | --- | --- | --- | --- |
| **Population** | **Segment 1** | **Segment 2** | **median[IQR] 1 (%)** | **median[IQR] 2 (%)** | **p-value** |
| **Lower arm** | AB | BPI | 19.5 [4.4] | 100.0 [0.0] | 0.0015 |
|  | AB | TR | 19.5 [4.4] | 17.7 [1.9] | 0.7389 |
|  | BPI | TR | 100.0 [0.0] | 17.7 [1.9] | 0.0062 |
| **Upper arm** | AB | BPI | 17.5 [3.3] | 39.6 [28.7] | 0.0339 |
|  | AB | TR | 17.5 [3.3] | 18.8 [0.9] | 0.8415 |
|  | BPI | TR | 39.6 [28.7] | 18.8 [0.9] | 0.0679 |
| **Shoulder** | AB | BPI | 18.1 [3.8] | 19.5 [3.3] | 0.8137 |
|  | AB | TR | 18.1 [3.8] | 17.6 [1.3] | 0.3861 |
|  | BPI | TR | 19.5 [3.3] | 17.6 [1.3] | 0.2733 |

**7.3. Tracking task**

| **Experiment 3, Correlation: Comparisons between participants** | | | | | |
| --- | --- | --- | --- | --- | --- |
| **Population** | **Segment 1** | **Segment 2** | **median[IQR] 1 (%)** | **median[IQR] 2 (%)** | **p-value** |
| BPI | Visual | Lower arm | 92.2 [3.1] | 0.0 [0.0] | 0.0625 |
|  | Visual | Upper arm | 92.2 [3.1] | 44.9 [17.2] | 0.0625 |
|  | Visual | Shoulder | 92.2 [3.1] | 68.1 [25.6] | 0.0625 |
|  | Lower arm | Upper arm | 0.0 [0.0] | 44.9 [17.2] | 0.0625 |
|  | Lower arm | Shoulder | 0.0 [0.0] | 68.1 [25.6] | 0.0625 |
|  | Upper arm | Shoulder | 44.9 [17.2] | 68.1 [25.6] | 0.1250 |
| TR | Visual | Lower arm | 93.7 [7.0] | 74.8 [29.0] | 0.0625 |
|  | Visual | Upper arm | 93.7 [7.0] | 81.2 [27.1] | 0.0625 |
|  | Visual | Shoulder | 93.7 [7.0] | 75.5 [38.4] | 0.1250 |
|  | Lower arm | Upper arm | 74.8 [29.0] | 81.2 [27.1] | 0.4375 |
|  | Lower arm | Shoulder | 74.8 [29.0] | 75.5 [38.4] | 0.6250 |
|  | Upper arm | Shoulder | 81.2 [27.1] | 75.5 [38.4] | 0.1250 |
| **Experiment 3, Error: Comparisons between participants** | | | | | |
| **Population** | **Segment 1** | **Segment 2** | **median[IQR] 1** | **median[IQR] 2** | **p-value** |
| BPI | Visual | Lower arm | 0.2 [0.0] | 0.0 [0.0] | 0.0625 |
|  | Visual | Upper arm | 0.2 [0.0] | 0.4 [0.0] | 0.0625 |
|  | Visual | Shoulder | 0.2 [0.0] | 0.4 [0.1] | 0.0625 |
|  | Lower arm | Upper arm | 0.0 [0.0] | 0.4 [0.0] | 0.0625 |
|  | Lower arm | Shoulder | 0.0 [0.0] | 0.4 [0.1] | 0.0625 |
|  | Upper arm | Shoulder | 0.4 [0.0] | 0.4 [0.1] | 0.3125 |
| TR | Visual | Lower arm | 0.2 [0.1] | 0.3 [0.2] | 0.0625 |
|  | Visual | Upper arm | 0.2 [0.1] | 0.3 [0.1] | 0.0625 |
|  | Visual | Shoulder | 0.2 [0.1] | 0.3 [0.2] | 0.1250 |
|  | Lower arm | Upper arm | 0.3 [0.2] | 0.3 [0.1] | 0.1875 |
|  | Lower arm | Shoulder | 0.3 [0.2] | 0.3 [0.2] | 0.4375 |
|  | Upper arm | Shoulder | 0.3 [0.1] | 0.3 [0.2] | 0.3125 |
| **Experiment 3, Delay: Comparisons between participants** | | | | | |
| **Population** | **Segment 1** | **Segment 2** | **median[IQR] 1 (ms)** | **median[IQR] 2 (ms)** | **p-value** |
| BPI | Visual | Lower arm | 42.5 [4.6] | 0.0 [0.0] | 0.0625 |
|  | Visual | Upper arm | 42.5 [4.6] | 71.6 [61.2] | 0.0625 |
|  | Visual | Shoulder | 42.5 [4.6] | 58.1 [25.4] | 0.1250 |
|  | Lower arm | Upper arm | 0.0 [0.0] | 71.6 [61.2] | 0.0625 |
|  | Lower arm | Shoulder | 0.0 [0.0] | 58.1 [25.4] | 0.0625 |
|  | Upper arm | Shoulder | 71.6 [61.2] | 58.1 [25.4] | 0.8125 |
| TR | Visual | Lower arm | 49.0 [6.6] | 58.6 [25.5] | 0.4375 |
|  | Visual | Upper arm | 49.0 [6.6] | 49.4 [17.2] | 1.0000 |
|  | Visual | Shoulder | 49.0 [6.6] | 56.7 [19.5] | 0.4375 |
|  | Lower arm | Upper arm | 58.6 [25.5] | 49.4 [17.2] | 0.0625 |
|  | Lower arm | Shoulder | 58.6 [25.5] | 56.7 [19.5] | 0.6250 |
|  | Upper arm | Shoulder | 49.4 [17.2] | 56.7 [19.5] | 0.1875 |

| **Experiment 3, Correlation: Comparisons between populations** | | | | | | |
| --- | --- | --- | --- | --- | --- | --- |
| **Population** | | **Segment 1** | **Segment 2** | **median[IQR] 1 (%)** | **median[IQR] 2 (%)** | **p-value** |
| **Visual** | | AB | BPI | 92.0 [1.9] | 92.2 [3.1] | 0.9468 |
|  |  | AB | TR | 92.0 [1.9] | 93.7 [7.0] | 0.7389 |
|  |  | BPI | TR | 92.2 [3.1] | 93.7 [7.0] | 1.0000 |
| **Lower arm** | | AB | BPI | 70.2 [12.3] | 0.0 [0.0] | 0.0027 |
|  |  | AB | TR | 70.2 [12.3] | 74.8 [29.0] | 0.5485 |
|  |  | BPI | TR | 0.0 [0.0] | 74.8 [29.0] | 0.0625 |
| **Upper arm** | | AB | BPI | 70.2 [10.1] | 44.9 [17.2] | 0.0041 |
|  |  | AB | TR | 70.2 [10.1] | 81.2 [27.1] | 0.5485 |
|  |  | BPI | TR | 44.9 [17.2] | 81.2 [27.1] | 0.0625 |
| **Shoulder** | | AB | BPI | 64.4 [14.7] | 68.1 [25.6] | 0.6407 |
|  |  | AB | TR | 64.4 [14.7] | 75.5 [38.4] | 0.6407 |
|  |  | BPI | TR | 68.1 [25.6] | 75.5 [38.4] | #ÜBERLAUF! |
|  |  |  |  |  |  |  |
| **Experiment 3, Error: Comparisons between populations** | | | | | | |
| **Population** | | **Segment 1** | **Segment 2** | **median[IQR] 1** | **median[IQR] 2** | **p-value** |
| **Visual** | | AB | BPI | 0.2 [0.0] | 0.2 [0.0] | 0.3861 |
|  |  | AB | TR | 0.2 [0.0] | 0.2 [0.1] | 0.6407 |
|  |  | BPI | TR | 0.2 [0.0] | 0.2 [0.1] | 0.6250 |
| **Lower arm** | | AB | BPI | 0.3 [0.1] | 0.0 [0.0] | 0.0027 |
|  |  | AB | TR | 0.3 [0.1] | 0.3 [0.2] | 0.9468 |
|  |  | BPI | TR | 0.0 [0.0] | 0.3 [0.2] | 0.0625 |
| **Upper arm** | | AB | BPI | 0.3 [0.1] | 0.4 [0.0] | 0.0027 |
|  |  | AB | TR | 0.3 [0.1] | 0.3 [0.1] | 0.7389 |
|  |  | BPI | TR | 0.4 [0.0] | 0.3 [0.1] | 0.0625 |
| **Shoulder** | | AB | BPI | 0.3 [0.0] | 0.4 [0.1] | 0.3861 |
|  |  | AB | TR | 0.3 [0.0] | 0.3 [0.2] | 0.9468 |
|  |  | BPI | TR | 0.4 [0.1] | 0.3 [0.2] | 0.3125 |
|  |  |  |  |  |  |  |
| **Experiment 3, Delay: Comparisons between populations** | | | | | | |
| **Population** | | **Segment 1** | **Segment 2** | **median[IQR] 1 (ms)** | **median[IQR] 2 (ms)** | **p-value** |
| **Visual** | | AB | BPI | 44.4 [7.4] | 42.5 [4.6] | 0.2571 |
|  |  | AB | TR | 44.4 [7.4] | 49.0 [6.6] | 0.5485 |
|  |  | BPI | TR | 42.5 [4.6] | 49.0 [6.6] | 0.3125 |
| **Lower arm** | | AB | BPI | 47.6 [9.1] | 0.0 [0.0] | 0.0027 |
|  |  | AB | TR | 47.6 [9.1] | 58.6 [25.5] | 0.3861 |
|  |  | BPI | TR | 0.0 [0.0] | 58.6 [25.5] | 0.0625 |
| **Upper arm** | | AB | BPI | 46.7 [6.0] | 71.6 [61.2] | 0.0388 |
|  |  | AB | TR | 46.7 [6.0] | 49.4 [17.2] | 0.9468 |
|  |  | BPI | TR | 71.6 [61.2] | 49.4 [17.2] | 0.1250 |
| **Shoulder** | | AB | BPI | 48.4 [13.2] | 58.1 [25.4] | 0.3861 |
|  |  | AB | TR | 48.4 [13.2] | 56.7 [19.5] | 0.9468 |
|  |  | BPI | TR | 58.1 [25.4] | 56.7 [19.5] | 0.8125 |
