# Supplementary material for: Impact of transradial amputation and brachial plexus injury on vibrotactile sensation capacity of the upper extremity
Source: J Neuroeng Rehabil. 2026 May 16;23:175. doi: 10.1186/s12984-026-02001-x (PMC13220424; doi:10.1186/s12984-026-02001-x)
Supplement: Supplementary file 2 — Supplementary Material 2. [file 12984_2026_2001_MOESM2_ESM.docx]

1. Supplementary Materials

**Supplementary Materials Table S1.** Descriptive statistics and effect sizes for between-group comparisons. Median values [interquartile range] and 95 % confidence intervals (CIs) are shown for the Transradial (TR) and Brachial Plexus Injury (BPI) groups in each tested segment and outcome measure. Cliff’s Δ [95 % CI] quantifies the magnitude and direction of the difference between each patient group and the able-bodied benchmark, where positive values indicate higher values in the patient group and negative values indicate higher values in the benchmark. Δ ≈ 0 denotes negligible difference, |Δ| ≥ 0.33 a medium effect, and |Δ| ≥ 0.47 a large effect.


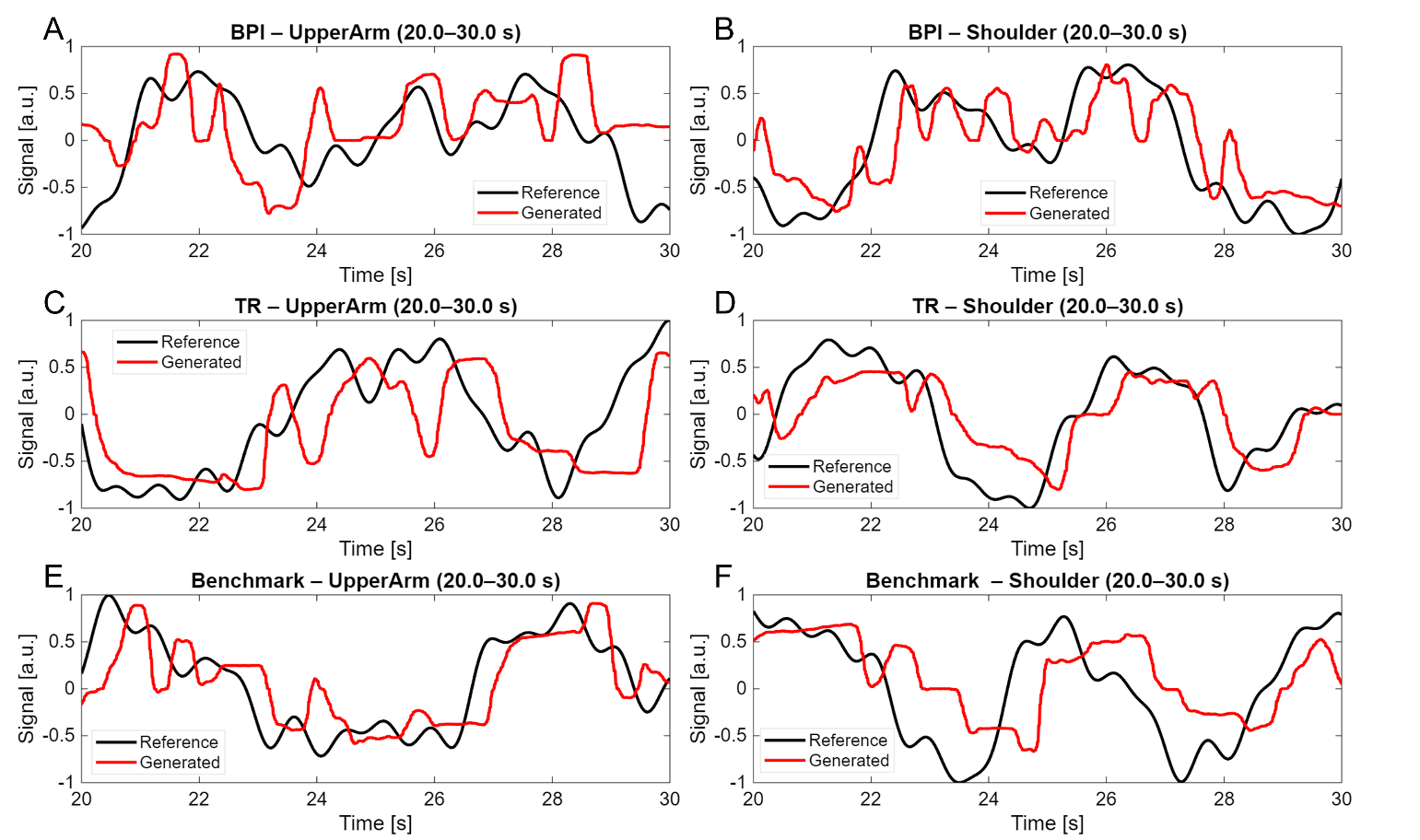


**Supplementary Materials Figure S1.** Exemplary compensatory tracking trajectories for persons with brachial plexus injury (BPI; A-B), persons with transradial amputation (TR; C-D), and healthy nondisabled participants (Benchmark; E-F). Shown are 10-s excerpts (20-30 s) of the reference trajectory (black) and the generated joystick signal (red) for the upper arm (left column) and shoulder (right column) segments. All signals are normalized to the range [-1, 1]. TR and Benchmark participants show largely overlapping tracking behavior, with similar timing and correction dynamics. In contrast, BPI participants exhibit more prolonged deviations from the reference during phases requiring corrective adjustments, suggesting reduced stabilization efficiency.
